# Long-term memory speeds encoding, independently of attention

**DOI:** 10.3758/s13423-026-02998-1

**Published:** 2026-09-08

**Authors:** Abigail C. Wyllie, Claudia C. von Bastian, Alon Zivony

**Affiliations:** School of Psychology, University of Sheffield, Sheffield, UK

**Keywords:** Working memory, Familiarity, Long term memory, Selective attention

## Abstract

Recent studies suggest that familiar objects benefit from faster working memory (WM) encoding – the transformation of fragile perceptual representations into durable WM representations. This observation led contemporary WM models to postulate a long-term memory (LTM) “template-matching” mechanism, which speeds standard encoding processes. However, faster encoding may also be caused by higher attentional priority for familiar objects. In the current study, we examined whether LTM template matching can be dissociated from selective attention. We used a Rapid Serial Visual Presentation task and a manipulation previously shown to have no effect on attention. While the selection cue remained constant throughout the experiment, targets were either English or Hebrew letters, presented to native English-speakers and Hebrew-speakers. In Experiments 1 and 2, when the target was one of 16 letters, participants were more accurate when reporting target letters from their (familiar) native language relative to non-native language. In Experiment 2, the target was preceded by peripheral distractors which resulted in an “attentional blink” effect. Although a familiarity benefit was present for target accuracy, familiar and unfamiliar distractors produced an attentional blink effect of equal magnitude, demonstrating equal efficacy in capturing attention. In Experiment 3, a familiarity effect emerged even when the target language was blocked. This shows that the familiarity effect observed in Experiments 1 and 2 cannot be attributed to a response bias in favour of reporting one’s own language. Overall, the current study provides robust evidence that LTM template-matching speeds encoding of familiar items, independently of attention.

**Supplementary Information:**

The online version contains supplementary material available at https://doi.org/10.3758/s13423-026-02998-1.

## Introduction

Working memory (WM) is a critical cognitive system that retains important information in an active state, thereby enabling complex cognitive processes and computations that rely on this information. Substantial research efforts have been dedicated to better understanding how WM temporarily stores and manipulates visual information (for reviews, see Baddeley, 2012; Chai et al., 2018). In comparison, relatively little study has been dedicated to WM encoding – the transformation of fragile perceptual representations into durable WM representations (Cappiello & Zhang, 2016; Cotton & Ricker, 2022; Vogel et al., 2006). Some models suggest that encoding is interlinked with selective attention. These models assume that once sensory information is attended, its encoding is inevitable (e.g., Wolfe, 2021). However, recent research suggests that factors other than attention may determine encoding. In particular, long-term memory (LTM) has been implicated in gating WM access for attended stimuli (e.g., Brogaard & Sørensen, 2024).

Perception and memory are profoundly shaped by our accumulated knowledge and experiences. A clear example of this is the interplay between visual perception and familiarity. In the context of visual cognition, an object is familiar if one has substantial prior experience with it (i.e., it is stored in LTM). A considerable amount of research, spanning decades, has shown that familiar objects are perceived better than unfamiliar objects (Christie & Klein, 1995; Krueger, 1975; Ramon & Gobbini, 2017). More recently, researchers have examined how familiarity affects short-term memory and WM performance.

The role of familiarity on memory is studied using one of two methods. The first involves training participants with novel stimuli until they are encoded into LTM (e.g., Blalock, 2015; Mizrak & Oberauer, 2022). This induced familiarity allows for a high degree of experimental control, as it manipulates the amount of experience a person has with the stimuli. The second method utilises pre-existing expertise with certain kinds of stimuli (e.g., Loaiza et al., 2025). This existing knowledge usually arises from years of experience, resulting in robust LTM representations which cannot be formed in a standard experimental paradigm. Notable examples of this methodology use stimuli such as letters or words in native versus non-native languages (e.g., Ngiam et al., 2019), famous versus unknown faces (e.g., Jackson & Raymond, 2008), or even familiar versus unfamiliar cartoon characters (Xie & Zhang, 2017). Collectively, these studies showed that familiarity is a critical modulator of the amount of information that can be stored and maintained in WM.

Familiar objects have also been found to be encoded more quickly than unfamiliar objects. For example, Xie and Zhang (2017, 2018) employed a modified change detection task with masking to assess the time course of WM encoding. Participants briefly viewed a memory array, followed by a mask at varying stimulus-onset asynchronies (SOAs) designed to disrupt visual sensory representations not yet consolidated into WM. Using this method, Xie and Zhang concluded that familiarity increased WM encoding speed, a conclusion supported by Li et al. (2020) and Ngiam et al. (2019). Recent WM models have incorporated the familiarity benefit to encoding as an example of the selectivity of WM encoding. These models suggest WM encoding speed is modulated by a “template-matching” process (Brogaard & Sørensen, 2024) that compares sensory information to existing LTM representations. These models postulate various mechanisms (Hedayati et al., 2022; Mizrak & Manohar, pre-print; Ngiam, 2024) which allow a matched stimulus to undergo a less rigorous perceptual process prior to encoding. For example, template matching may allow familiar stimuli to bypass standard binding processes (Ngiam, 2024), reduce the processing threshold required for encoding a familiar item (Zivony & Eimer, 2024), or produce more compact WM representations for familiar stimuli to be mapped onto during encoding (Hedayati et al., 2022). Differentiating between different template-matching accounts has important implications for understanding WM encoding. However, a fundamental assumption these accounts share has not yet been robustly tested, that is, that LTM expedites encoding independently from attention.

Template-matching accounts postulate two co-existing yet separate processes that promote the encoding of familiar objects. First, these models recognise that familiarity can capture and engage attention (Christie & Klein, 1995; Jessen & Rodway, 2010; Risko et al., 2011), which can improve bottom-up processing and therefore encoding of familiar objects. Hence, attention can be understood as a factor that “pushes” information forward from sensory memory to WM. At the same time, template-matching accounts also postulate a separate reversed-hierarchy process whereby pre-existing LTM representations “pull” sensory information into WM, allowing encoding of even weakly processed stimuli. This reversed-hierarchy process is assumed to be distinct from any attentional modulation automatically granted to familiar objects. However, since attentional prioritization can also lead to faster encoding into WM (Reinitz, 1990), it is unclear whether findings that support the template-matching hypothesis should be attributed to attention instead.

The current study tests the key, but yet untested, assumption shared between template-matching accounts: that template matching is a process distinct from attentional modulations granted to familiar objects. A strong test of this assumption requires a dissociation between familiarity effects on WM encoding and familiarity effects on selective perceptual attention. If this dissociation cannot be demonstrated, it would suggest that familiarity effects are produced by attentional modulation, rather than a distinct WM encoding mechanism, undermining the template-matching hypothesis. Therefore, the current study is a critical stepping-stone towards any future research aimed at pinpointing the exact mechanism underlying template matching.

## The current study

Dissociating selective attention from other selective processes that affect WM encoding is a challenging task. Recently, Zivony and Eimer (2024) developed a task meant to address this challenge. They used a Rapid Serial Visual Presentation (RSVP) task where accuracy depends on quickly encoding the target before it is overridden by a distractor (Wyble et al., 2011). Target detection was based solely on low-level defining features (e.g., unique colour), yet participants were more likely to correctly report the target’s identity if it was from a high-probability alphanumeric category (digit vs. letter). Since the target’s alphanumeric category was a high-level feature, and ineffective in differentiating between the target and distractors, it was argued that alphanumeric category should have had no control over attention. In a following experiment, distractors that shared the target-defining feature were presented laterally prior to the target. This resulted in attentional capture and a temporary reduction in overall accuracy – an attentional blink (AB) effect (Folk et al., 2002; Zivony & Lamy, 2014). Importantly, attentional capture and engagement was not modulated by expectations: distractors reduced target accuracy equally (i.e., an equal AB effect), whether they were from the more probable (expected) or less probable (unexpected) target category. This study therefore demonstrated that, under certain conditions, expectations could improve WM encoding independently of attentional deployment.

In the current study, we employed the same rationale as Zivony and Eimer (2024) to test whether template matching expedites WM encoding, independently of attention. The target letter appeared in one of two languages matched for perceptual complexity: English and Hebrew. We examined whether greater familiarity with a target’s language (i.e., native language vs. non-native language) resulted in higher accuracy in an RSVP task. Crucially, Experiment 2 examined whether familiarity effects emerge independently of attentional capture and engagement by employing an AB design similar to Zivony and Eimer (2024). To preview the results, we found clear evidence for a familiarity effect, but no evidence that familiarity modulated attentional capture, providing robust support for the LTM template-matching hypothesis.

## Experiment 1

### Method

In this and the following experiments, we compared the processing of uppercase English letters to the processing of Hebrew letters among native English speakers (Experiments 1–3) and native Hebrew speakers (Experiment 1). The main reason to choose these languages was that a previous study found that Hebrew letters are comparable to English letters in both complexity and how efficiently they are perceived (Pelli et al., 2006). This suggests that differences found in memory for these stimuli are unlikely to reflect low-level differences. A secondary reason was that Hebrew is not commonly used worldwide, which reduces the likelihood that English-speaking participants would have any familiarity with Hebrew letters. It should be noted that Hebrew and English are read from different directions (left-to-right vs. right-to-left). Differences in reading direction have been shown to affect performance in similar RSVP tasks (Ransley et al., 2018). However, this should not have affected the present experiments, as we relied on a single, centrally presented, RSVP stream.

#### Ethics

All methods used in this experiment, and subsequent experiments, were approved by the Ethics Committee at the University of Sheffield’s School of Psychology.

#### Sample size selection

We used a task similar to the one used in Zivony and Eimer (2024). In that study, the target was either a cued letter or digit, and the alphanumerical category of the target was manipulated to be either expected or unexpected. Accuracy was higher when the target was from the expected alphanumeric category. Since we expected an effect of similar size, we used the effect observed in that study (η^2^_p_ =.47) to justify our sample size. Using G*Power, we calculated that the required sample size to observe an effect of this magnitude with 80% power is *N* = 12. To allow for the detection of smaller effects, we used a sample of *n* = 20 per group. Since the experiment included a native English-speaking group and a native Hebrew-speaking group, we recruited an overall sample of *N* = 40.

#### Participants

Forty participants took part in the study: 20 native Hebrew speakers and 20 native English speakers. Two participants were removed because of technical difficulties (see below), but were replaced to achieve the preferred sample size of *n* = 20 for both groups. Participants were recruited through Prolific. To ensure we obtained the correct participant language profile, we recruited participants using the pre-obtained demographic data from Prolific. The native Hebrew-speaking volunteers consisted of 12 men and eight women, (*M*_age_ = 35 years, *SD* = 12.2). They were residents of Israel (16), the USA (2), Canada (1), and the UK (1). The native English-speaking volunteers comprised 13 men and seven women (*M*_age_ = 35.2 years, *SD* = 14.2). They were residents of the UK (11), South Africa (5), the USA (2), Portugal (1), and Italy (1). Participants received £3.30 (£10/h) for completing the study. No other demographic information was collected.

#### Apparatus

The experiment was conducted using participants’ own computers, who accessed and downloaded the experiment via the E-Prime Go cloud service. Subjects were asked to sit approximately 60 cm from the screen (approximately an arm’s length), in a quiet and distraction-free environment, and complete the task in one sitting within 35 min. Manual responses were given via a standard keyboard.

#### Design, procedure, and stimulus

All stimuli sizes were calculated in visual angles based on the participants’ self-reported monitor size and an assumed distance of 60 cm from the screen. If participants did not know their monitor size, they were directed to a website that calculated it for them.

**Fig. 1:**
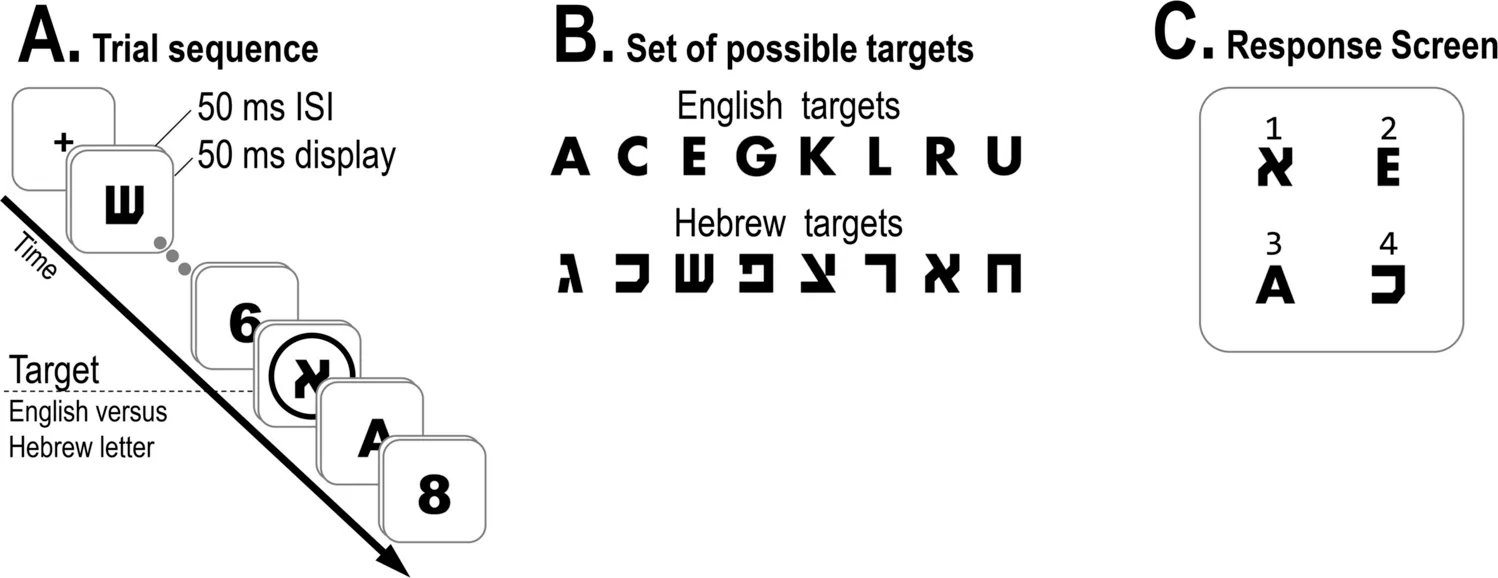
Illustration of the paradigm used in Experiment 1. The task was to find a target character inside a circle embedded in a Rapid Serial Visual Presentation (RSVP) stream of digits and letters (**A**). The target was either an English letter or a Hebrew letter (**B**). Hebrew letters are presented below matched English letters. After the RSVP, participants reported the identity of the target (**C**)

Figure 1 illustrates the task. Participants were asked to report, as accurately as possible, the identity of a target letter that appeared inside the selection feature, which was a circle cue (0.8° radius). These targets were presented at unpredictable temporal positions in an RSVP stream that appeared in the centre of the screen. Manual responses were executed without time pressure at the end of each trial.

Each trial began with the presentation of a fixation cross (a grey 0.2° × 0.2° “ + ” sign at the centre of the screen). After 500 ms, the RSVP stream appeared. Each frame appeared for 50 ms with a 50 ms interstimulus interval (ISI). The software used in this study allows for a high degree of control over presentation rates and tracking of refresh rates. Participants whose computers could not produce this presentation rate were excluded from the sample.

The target appeared unpredictably in the sixth, eighth, or tenth frame within the RSVP stream with equal probability and was followed by two additional distractors (Fig. 1A). Therefore, the length of each RSVP trial was between eight and 12 frames. All characters in the RSVP streams were selected from a set of eight digits (2–9), a set of eight English letters, and a set of eight Hebrew letters (Fig. 1B). The digits were printed in Arial Black font, English letters in Aharoni font, and Hebrew letters in Guttman Haim. The specific English and Hebrew letters used here were chosen based on their perceptual similarities to one another (Fig. 1B). Moreover, Hebrew letters were subtly stretched in order to match the proportions of the English letters. All alphanumeric characters were 1.3° in height, were written in black, and were presented on a white background.

Items in the RSVP were drawn without replacement from the possible set of characters. The character selection was random except for the following restrictions: half of the items in the RSVP were digits and the rest were letters. Half of the letters in the RSVP were Hebrew letters, and the other half were English letters. The target was always a letter; this letter was drawn from the set of Hebrew letters for 50% of the trials and from the English letter set for the rest. This target was always preceded by a digit and followed by a letter. Following Zivony and Eimer (2024), this design was chosen to allow for post-target distractor intrusion reports and to prevent pre-target reports. Note that while the target was always preceded by a digit, the presence of a digit was not predictive of the target’s appearance, as digits frequently appeared throughout the RSVP stream. The last item in the RSVP stream was always a digit.

After the RSVP stream, the response screen appeared, organised in a 2 × 2 matrix centred in the middle of the screen. The matrix contained two Hebrew letters and two English letters. One of the letters was the target, another was the post-target letter, and two were foils that did not appear in the RSVP. The location of letters was randomized. The letters appeared in the centre of the matrix cells. The centre-to-centre distance between cells was 2.5°. A number from 1 to 4 appeared above each of the letters to indicate the relevant response key (Fig. 1C).

Participants reported the target by pressing the relevant key on their keyboard. Once pressed, a (0.8° radius) circle appeared for 200 ms around the selected character to provide participants with visual feedback that their response was registered. Following feedback, a blank screen appeared for 800 ms before a new trial started. The experiment included 10 practice trials followed by 96 experimental trials, divided into two 48-trial blocks.

Finally, following the experiment, participants reported whether they were native English or Hebrew speakers. They also indicated whether they were proficient in speaking, writing, and reading English and/or Hebrew. Participants in the native English-speaking group were proficient in speaking, writing, and reading English but not Hebrew. Participants in the native Hebrew-speaking group were proficient in both languages.

#### Open data

All the data and materials necessary to reproduce the experiment and analyses are available online at: https://doi.org/10.6084/m9.figshare.31026025. None of the study’s analyses were preregistered.

#### Analysis

The main analysis examined the effect of familiarity on performance. Average accuracy rates were entered into a repeated-measures ANOVA with target language (English vs. Hebrew) and native-language group (English speakers vs. Hebrew speakers) as the independent factors. However, some tests reported in this experiment, and the following experiments, include the interpretation of null results. Therefore, statistical tests were supplemented with corresponding Bayes factors: statistically non-significant tests and statistically significant tests were supplemented with a Bayes factor in favour of the null hypothesis (*BF*_01_) and alternative hypothesis (*BF*_10_), respectively. All tests were conducted using the anovaBF and lmBF functions from the BayesFactor package in R (Morey et al., 2018). As recommended by van Doorn et al. (2023), we used the “maximal” model (i.e., the model with both participant intercepts and effect slopes as random effects) to evaluate our effects. Bayes factors associated with a two-way interaction were calculated by dividing the Bayes factor associated with the full model by the Bayes factor associated with the model that includes only the two main effects. Since we had no a priori expectations regarding these effects, we used the default medium prior (*r* = 0.5), yet in all experiments we obtained similar results with wider priors (*r* = 0.707 or *r* = 1.0). We considered a *BF* > 3 to provide unambiguous evidence, and a *BF* > 10 to provide substantial evidence for the relevant (null or alternative) hypothesis.

Sensitivity analysis was conducted to ensure that our results were not caused by any outlier participants. Specifically, Zivony et al. (2026) recommended, for analyses relating to individual differences, to exclude participants with a “guess” rate (i.e., reporting the identity of neither the target nor nearby distractors) of over 30%. In this study, only one participant met this criterion. While the exclusion recommendation related to correlational analyses (not within-subject comparisons), we nevertheless conducted all the analyses whilst excluding this participant. This analysis revealed that all the conclusions remained the same.

### Results

**Fig. 2:**
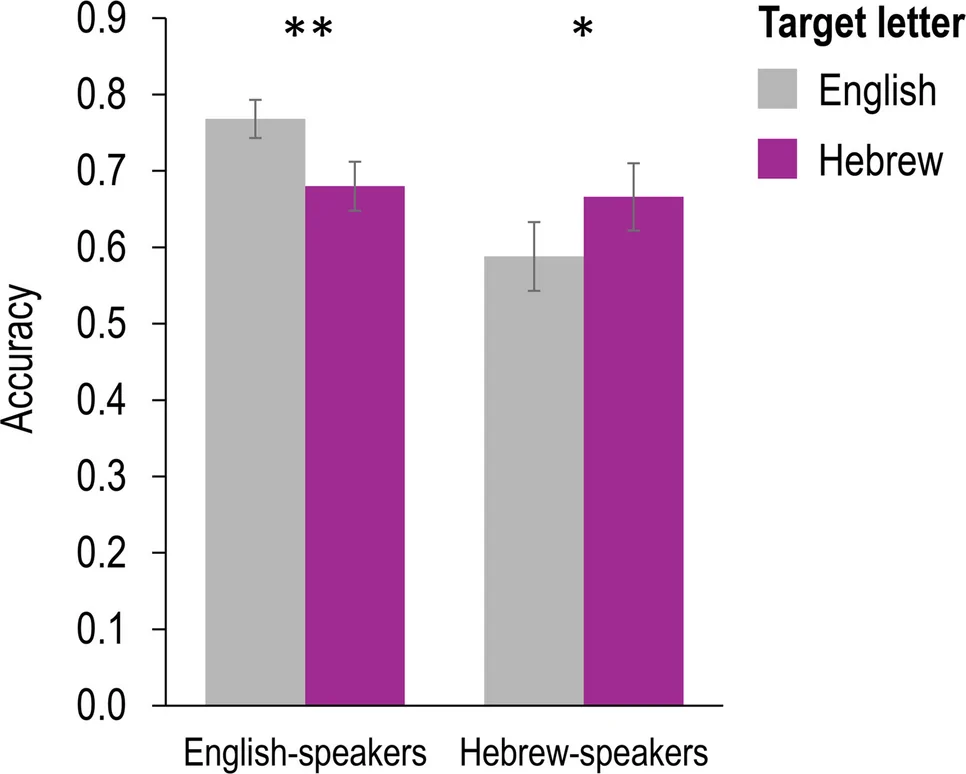
Mean accuracy in Experiment 1 as a function of target letter language (English vs. Hebrew) and participant language (English-speakers vs. Hebrew-speakers). Error bars reflect one within-subject standard error. * *p* <.05, ** *p* < 01

The main effects of native language, *F*(1,38) = 3.98, *p* =.053, ${\eta}_{p}^{2}$ =.10, *BF*_*01*_ = 1.65, and target language, *F*(1,38) = 0.06, *p* =.81 ${\eta}_{p}^{2}$ =.002, *BF*_*01*_ > 100, were non-significant. Importantly, their interaction was significant, *F*(1,38) = 16.40, *p* <.001, ${\eta}_{p}^{2}$=.30, *BF*_*10*_ > 100 (Fig. 2). For native English speakers, accuracy was higher when the target was in English versus Hebrew (*M* = 76.8% vs. 68.0%), while the opposite was true for native Hebrew speakers (*M* = 58.8% vs. 66.6%). Simple-effects analysis confirmed that both differences were significant, *F*(1,38) = 9.21, *p* =.004, ${\eta}_{p}^{2}$ =.20, *BF*_*10*_ > 100, and *F*(1,38) = 7.25, *p* =.01, ${\eta}_{p}^{2}$=.16, *BF*_*10*_ = 67.28, respectively.

### Discussion

Experiment 1 showed that participants are more likely to encode letters from their native language. Therefore, the familiarity effect was not caused by perceptual differences between English and Hebrew letters. Moreover, given that the Hebrew-speaking participants were all proficient in English to some degree, whereas the English-speaking participants had no Hebrew proficiency, the observed effects cannot be due to mere exposure to the stimuli, but rather reflect differences in long-term expertise.

We also note a trend towards overall lower accuracy in the Hebrew-speaking group compared to the English-speaking group (72.3% vs. 62.7%). There are various post hoc reasons why this pattern could emerge. For example, Hebrew-speaking participants had to reject relatively familiar distractors in a way English speakers did not, thereby increasing overall task difficulty. However, since this trend was inconclusive and non-significant, we hesitate to draw strong conclusions from these findings.

The results observed in Experiment 1 suggest that a language-based familiarity effect on WM encoding can emerge even when participants search for another feature (the circle cue). This suggests that familiarity may affect WM encoding independently of attention. However, a stronger test for this dissociation requires a separate measure of attention.

## Experiment 2

In Experiment 2, we measured whether familiarity affects attentional capture and engagement using a variant of the distractor-based AB task from Zivony and Eimer (2024). Participants searched for a target in the middle of three RSVP streams. On some trials, two distractors appeared simultaneously in the two lateral streams prior to the target. These distractors were expected to capture and engage attention, thereby producing an AB effect (i.e., reduce accuracy of reporting a target that appears within 500 ms). Since AB effects only emerge following attentional engagement (Ophir et al., 2020), and since attentional engagement is affected by all preceding sustained attentional mechanisms (e.g., spatial, feature-based, temporal), any interaction between familiarity and attention should be expressed as differences in AB rates (for further discussion, see Zivony & Eimer, 2024). Therefore, if familiar distractors are attentionally prioritized, they should be more likely to capture and engage attention, thereby producing a larger AB effect and reduction in accuracy. In contrast, if familiar stimuli only benefit from LTM template matching, distractor familiarity should not modulate attentional capture and should produce AB effects of equal size.

### Method

#### Sample size selection

We based our sample size on the familiarity effect size in the English-speaking group in Experiment 1, ${\eta}_{p}^{2}$ =.35. Using G*Power, we calculated that the required sample size to observe an effect of this magnitude with 80% power is *N* = 18. To allow for a better comparison with Experiment 1, we used a sample of *N* = 20.

#### Participants

The sample included 12 men and eight women, with a mean age of 38.40 years (*SD* = 12.91). All participants were UK residents, native English speakers, and had no proficiency in Hebrew speaking, reading, or writing. Participants were recruited via Prolific and were paid £3.30 for their participation.

#### Apparatus, design, and stimuli

**Fig. 3:**
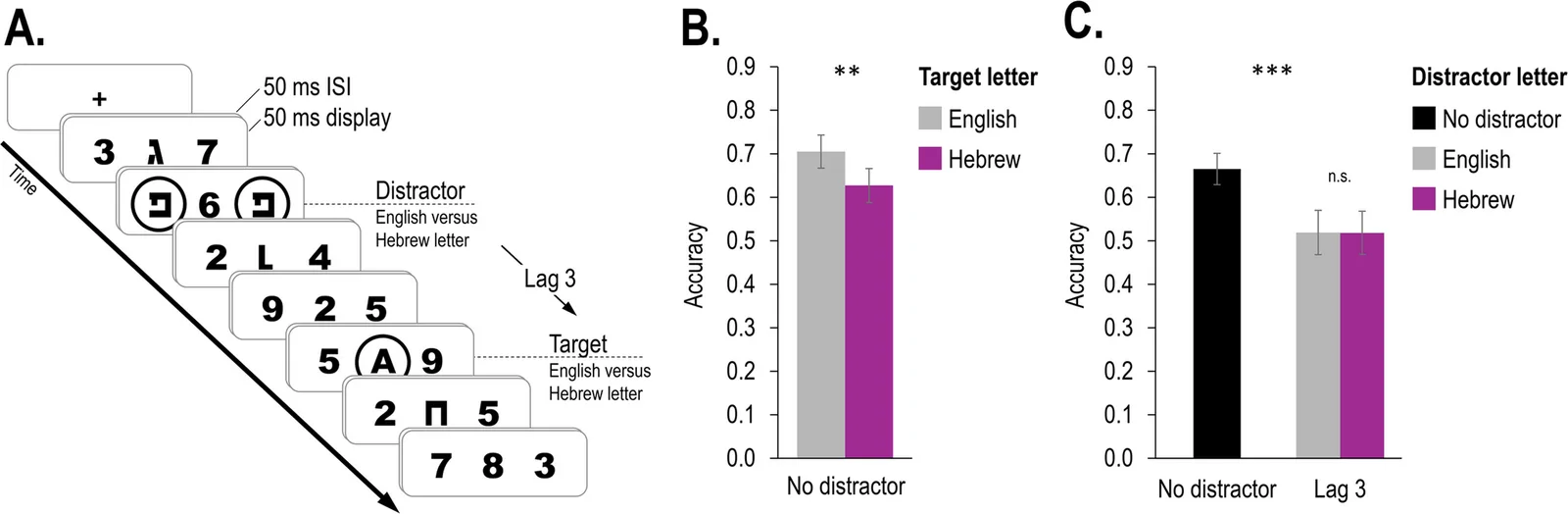
Illustration of the paradigm used in Experiment 2 and associated results. The task was to find a target character inside a circle (**A**), embedded in a central Rapid Serial Visual Presentation (RSVP) stream of digits and letters, and report its identity. The target was either a Hebrew letter or an English letter. On two-thirds of the trials, two cued distractors appeared three frames before the target. These distractors were either Hebrew letters or English letters. When no distractors were present, accuracy was higher for English targets relative to Hebrew targets (**B**). When distractors were present, accuracy was reduced to the same extent whether the distractors contained English letter distractors or Hebrew letter distractors (**C**). These results show that both distractors captured attention and reduced accuracy to the same extent

The apparatus, design, and stimuli were all identical to Experiment 1, except for the following changes (Fig. 3A). Participants were presented with three RSVP streams, one presented centrally, and two that appeared 2.5° to the left and right of fixation. The target could only appear on the central stream, and (like in Experiment 1) was a letter defined by a cue circle. The two lateral streams included mostly digits, drawn randomly and with replacement, with the sole restriction that the same digit could not appear in the same frame in the two streams or on the same stream in two subsequent frames. On two-thirds of the trials, one frame (the distractor frame) included two letters inside a circle in the lateral streams. These letters were equally likely to be Hebrew or English. The inclusion of two distractor RSVP streams ensured that attentional capture will not be affected by reading direction (Ransley et al., 2018). When the distractor frame appeared, it appeared three frames before the target (Lag 3). Participants were instructed to search for the target only in the central stream; they were also informed that irrelevant distractors would appear in the periphery, and that they should ignore these distractors. The experiment included 10 practice trials followed by 180 experimental trials, divided into three 60-trial blocks.

#### Analysis

In Experiment 2, we conducted two main analyses. The purpose of the first analysis was to replicate the familiarity effect observed in Experiment 1. For this reason, we excluded all trials where a distractor appeared. We then compared accuracy as a function of the target language (Hebrew letter vs. English letter). The second analysis was aimed to examine the effect of the peripheral distractors on accuracy. For these analyses, we collapsed accuracy data across the two target types and compared average accuracy when the distractor appeared at Lag 3 as function of the distractor type.

We also reasoned that if the familiarity effect observed in Experiment 1 relied on attention, then familiarity effects on accuracy and attentional capture should be correlated. That is, participants who showed a larger familiarity effect on target accuracy should also show a larger AB for familiar distractors. We therefore conducted an exploratory analysis to test this prediction. We calculated two measures for each participant. The familiarity effect on target accuracy was calculated as the difference between accuracy reporting the English letter and the Hebrew letter, and the familiarity effect on attentional capture was calculated as the difference in accuracy when the distractor was a Hebrew letter and when the distractor was an English letter. The Bayesian analysis was the same as in Experiment 1.

One participant showed a guess rate of above 30%. We therefore conducted a sensitivity analysis and repeated all analyses excluding this participant. These analyses revealed that all the conclusions remained the same.

### Results

#### Target familiarity

As with the English-speaking group in Experiment 1, accuracy was higher for English targets than Hebrew targets, *F*(1,19) = 8.18, *p* =.01, ${\eta}_{p}^{2}$ =.30, *BF*_*10*_ = 6.02 (Fig. 2B).

#### Distractor familiarity

Accuracy was lower when distractors appeared at Lag 3 relative to when no distractor appeared, *M* = 52.0% versus *M* = 66.5%, *t*(19) = 2.88, *p* =.01, *d* = 0.65, *BF*_*10*_ > 100, indicative of attentional capture and an AB-like effect. However, when distractors appeared at Lag 3, there was no difference in accuracy when the distractors were in English versus Hebrew, *M* = 51.9% versus *M* = 51.8%, *F*(1,19) = 0.001, *p* =.97, ${\eta}_{p}^{2}$ =.001, *BF*_*01*_ = 21.76 (Fig. 3B). Additional analysis showed this effect was not modulated by target familiarity, *F*(1,19) = 0.01, *p* =.92, ${\eta}_{p}^{2}$ <.001, *BF01* = 15.53. These results suggest attentional capture was not modulated by distractor familiarity.

#### Correlation analysis

The exploratory correlation analysis between familiarity effects on accuracy and attentional capture showed no evidence of a positive correlation, *r*(18) = -.12, *p* =.62, *BF*_*01*_ = 5.08.

### Discussion

Experiment 2 yielded three main findings. First, the familiarity effect observed in Experiment 1 was replicated for an English-speaking group. Second, as expected, peripheral distractors that matched the target-defining feature (a circle cue) automatically captured attention and produced an AB-like effect. Thirdly, and importantly, there was no difference in overall accuracy as a function of the letters inside the distractor cues. We also found no relationship between the benefit accrued by a familiar target and attentional capture by a familiar distractor. Altogether, this suggests that while letter familiarity affected WM encoding, it had no effect on selective attention.

## Experiment 3

It is possible that, rather than through LTM template matching, the observed familiarity effects emerged due to response bias. Specifically, when participants have to report a target from either their native language or a foreign language, they may prefer to report an item in their native language when unsure of the answer. Such bias would produce a systematic benefit whenever the target is from the participant’s native language and vice versa when it is not.

To test this alternative account, in Experiment 3 we limited the number of potential targets on a given block to two items. The two items could be either from the same language (both Hebrew or both English) or a mix of the two languages. If familiarity effects observed in Experiments 1 and 2 emerge only due to own-language response bias, then the effect should only be replicated in the mixed language blocks, where preferring an own-language response can affect results, and not in the same-language blocks.

### Method

#### Sample size selection

As in Experiment 2, we based our sample size on the likelihood of finding a familiarity effect in an English-speaking group, and therefore once again used a sample of *N* = 20.

#### Participants

The sample included 10 men and 10 women, with a mean age of 44.15 years (*SD* = 12.76). All participants were UK residents, native English speakers, and had no proficiency in Hebrew speaking, reading, or writing. Participants were recruited via Prolific and were paid £3.30 for their participation.

#### Apparatus, design, and stimuli

**Fig. 4:**
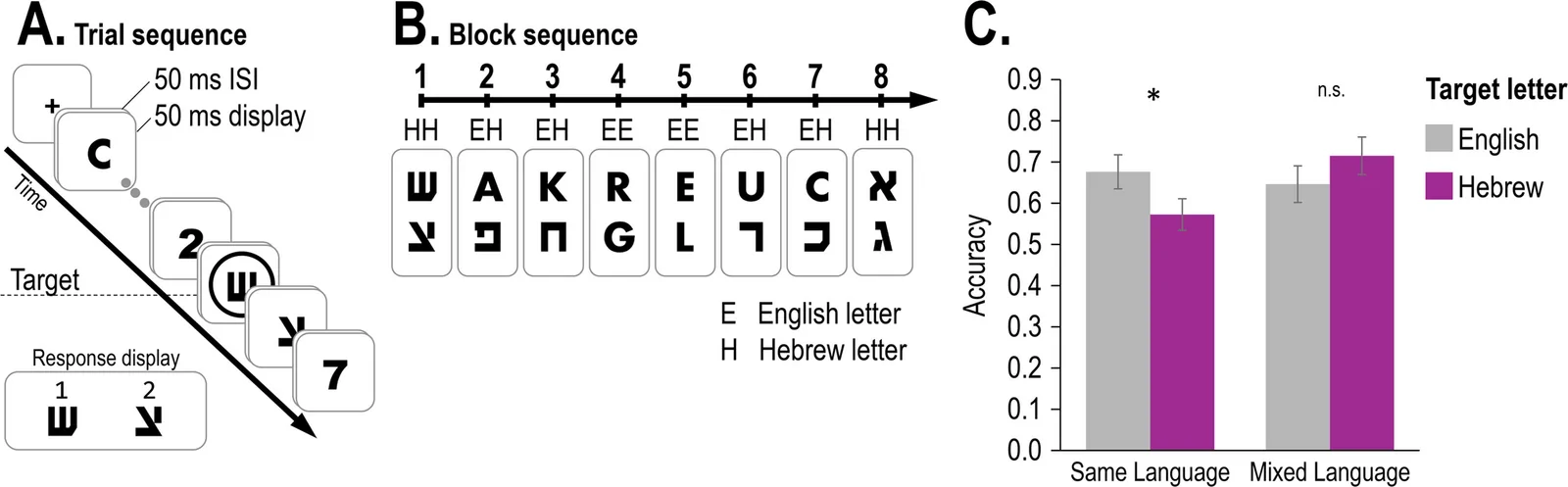
Illustration of the trial sequence (**A**) and the block sequence (**B**) used in Experiment 3 and associated results (**C**). Throughout each block of trials, the target could be one of two randomly selected targets. The items appeared in the target location or the post-target location (**A**). In four blocks, the two letters were a mix of an English letter and a Hebrew letter, in two blocks the two letters were both English letters, and in another two blocks the two letters were both Hebrew letters (**B**). The order of these blocks was randomised for each participant. Accuracy was higher for English letter targets than Hebrew letter targets, but only in the Same Language blocks, not Mixed Language blocks (**C**)

The apparatus, design, and stimuli were all identical to Experiment 1, except for the following changes. Target selection was based on a blocked design. In every task block, only two letters could be the target. The RSVP always included the two possible target letters, one inside the selection cue, and the other in the immediately following frame. Which item was the target and which item the post-target distractor was randomised for each trial, therefore maintaining equal probability of each target even though there were fewer options. The identity of these letters was chosen at random at the beginning of each block and then remained the same throughout (Fig. 4A). The response screen included only the two possible items. The rest of the RSVP stream consisted of randomly selected non-target letters and numbers. Therefore, the target category (whether English or Hebrew) was not enough to detect the target.

Participants completed eight blocks of 20 trials. At the beginning of each block, they were informed which two letters could be the targets on that block. The targets in four of the blocks were an English letter and a Hebrew letter, in two blocks the targets were two English letters, and in the other two blocks they were two Hebrew letters. Which letter appeared in which block was selected randomly without replacement for each participant, and the order of blocks was randomised (Fig. 4B). The practice block comprised 10 trials and always contained two English target letters.

#### Analysis

Accuracy was entered as a dependent variable to an ANOVA with Block Type (Mixed language vs. Same language) and Target language (English vs. Hebrew) as within-subject factors. In Experiment 3 there was no way to evaluate “guess” rates, as participants could only report the identity of the target or the post-target distractor. Therefore, we conducted sensitivity analyses by excluding the participant with the fastest reaction times and another participant who, in one block, reported the same target 20 times in a row. These analyses revealed that all the conclusions remained the same.

### Results

Accuracy as function of Block Type and Target Language are presented in Fig. 4C. The interaction between the two factors was significant, *F*(1,19) = 9.96, *p* =.005, ${\eta}_{p}^{2}$ =.34, *BF10* > 100. Therefore, we examined the effect of Target Language separately for Same Language blocks and Mixed Language blocks. In the Same Language block, accuracy was higher for English targets relative to Hebrew targets, *M* = 67.6% versus *M* = 57.3%, *t*(19) = 2.41, *p* =.026, *d* = 0.54. In contrast, in the Mixed Language blocks, accuracy was somewhat lower for English targets relative to Hebrew targets, *M* = 64.6% versus *M* = 71.5%, though this effect was not significant, *t*(19) = −1.52, *p* =.14, *d* = 0.34.

Finally, we compared accuracy between Experiment 3 and Experiments 1 and 2 (where the set-size of possible targets was 16 items). To do so, we aggregated data from English speakers from Experiment 1 and the no-distractor condition from Experiment 2. The difference between Experiments 3 and Experiments 1 and 2 was not significant, *M* = 70.1% versus *M* = 65.3%, *t*(57) = 1.08, *p* =.29, *d* = 0.29, *BF*_*01*_ = 5.88.

### Discussion

Experiment 3 revealed that own-language response bias cannot account for the familiarity effect observed in Experiments 1 and 2. Had it been, the familiarity effect should have been observed in Mixed Language blocks, where such bias can affect responses, but not in Same Language blocks. In fact, the opposite pattern emerged. Therefore, we conclude that the effects observed in Experiments 1 and 2 reflect true differences in encodability of familiar relative to unfamiliar objects, leading to differences in conscious perception.

How can an LTM template-matching account explain the absence of a familiarity effect in the Mixed Language block? A possible reason for this null effect relates to the restriction of the number of targets to two items. Specifically, it is possible that when participants have only two potential targets, they store these items in WM (Carlisle et al., 2011) as categorization templates (Zivony & Eimer, 2022). In turn, this should reduce the need to rely on LTM for search (Wolfe, 2020) and identification. In that case, any effects that rely on LTM activation may be attenuated (Bartsch & Oberauer, 2023; Mizrak & Oberauer, 2021), including familiarity effects. However, this speculation requires further research before any clear-cut conclusions can be reached about the dependency of LTM template matching on WM availability.

Finally, it is also noteworthy that despite the substantial reduction to set size, overall accuracy did not change relative to Experiments 1 and 2. This is comparable to similar RSVP experiments where set-size manipulations had no observable effect on overall accuracy (Zivony & Eimer, 2022). A likely explanation for this is that, in all experiments, targets were followed by reportable distractors, and most errors consisted of post-target distractor intrusions. Indeed, in Experiments 1 and 2, overall accuracy and intrusion rates sum up to approximately 94%, thereby limiting the potential for increased accuracy.

## General discussion

According to the template-matching hypothesis (Brogaard & Sørensen, 2024), items stored in LTM can expedite the encoding of matching sensory information into WM. A key challenge to the template-matching account lies in the difficulty in dissociating template matching from other processes that may produce similar effects, specifically selective attention. Automatic attentional prioritization for familiar items provides an alternative account for observed encoding benefits for familiar items. In this study, we employed an RSVP task to provide critical tests for this hypothesis. Observers searched for a single target, defined by an encircling selection cue, and had to report its identity. This target letter was drawn from one of two languages, the observers’ native language and another, less familiar, language.

The first prediction we tested was that LTM template matching should result in a familiarity benefit to encoding. In the first two experiments, native English speakers showed higher accuracy in reporting an English letter target relative to a Hebrew letter, and vice versa for native Hebrew speakers. These findings suggest that familiarity speeds up WM encoding, in line with previous studies that used the change detection task (Li et al., 2020; Ngiam et al., 2019; Xie & Zhang, 2017). In change detection tasks, participants attempt to encode multiple items concurrently. Encoding rate can therefore only be calculated indirectly, as a function of the number of objects encoded per unit of time. The current study extends previous studies by showing a familiarity benefit even when participants need to encode only a single item.

In the Online Supplementary Materials, we report additional analyses that support the conclusion that familiarity speeds WM encoding. It shows that the familiarity effect in Experiments 1 and 2 emerged more strongly when the target was followed by an unfamiliar distractor. This suggests that familiarity helps protect the target from perceptual competition from nearby distractors that may override it before it can be encoded. If both the target and distractor had an equal status in LTM, both have an increased likelihood of being encoded, thereby cancelling out the benefit to the target.

The second and main prediction we tested was that LTM template matching promotes encoding independently of attention. We used a task where the key familiarity manipulation was unlikely to affect attention. Specifically, since the target’s familiarity has no strategic benefit to the task, participants were expected to search for the target solely based on the simple feature that defines it (the encircling disk). Under such conditions, the familiarity of the target is unlikely to draw attention, and any familiarity benefit is unlikely to reflect superior attentional modulation. Importantly, in Experiment 2, we directly tested this by examining whether a familiar distractor item was more likely to capture attention. Despite clear evidence of a familiarity effect on target accuracy, we found that familiar and unfamiliar distractors were equally likely to capture attention and produce an attentional blink of equal magnitude.

Note that we do not suggest that familiarity affects processing solely via an attention-independent route. Indeed, all WM models that incorporate template matching also acknowledge that familiarity can affect attention. Rather, we merely conclude that attention-independent routes exist, and can be observed under experimentally controlled conditions. The exact mechanisms underlying LTM template matching are still unknown, and many different options have been postulated. The current study contributes to this effort by providing an experimental paradigm that controls for attentional processing. This is key for future efforts to clarify whether the mechanism underlying template matching is a separate binding process (Ngiam, 2024), compact WM representations (Hedayati et al., 2022), encoding despite limited sensory representation (Zivony & Eimer, 2024), or other theorised mechanisms (Mizrak & Manohar, pre-print).

Finally, the current study adds to a growing literature demonstrating selective effects on WM encoding that are not caused by attention (e.g., Cai et al., 2025; Wyble & Chen, 2017; Zivony & Eimer, 2022, 2024). Such effects cannot be easily explained by models that assume that attended objects should always be encoded (e.g., Wolfe, 2021). In contrast, these findings fit well with “diachronic” accounts of attention (Wyble et al., 2011; Zivony & Eimer, 2022). According to these accounts, attention should be understood as a modulatory process that gradually unfolds over time. These modulations enhance perceptual processing and therefore substantially increase the likelihood of WM encoding for modulated objects. However, these models postulate multiple mechanisms that can promote selectivity, operating alongside attention. Factors such as expectations or familiarity may affect perception both by affecting attention or via attention-independent routes. In other words, attention may be a critical but not the only cognitive factor that determines selection.

Selectivity is understood as a general principle at the heart of the perceptual process, which is supported by multiple interrelated cognitive mechanisms. We hope that by studying these mechanisms and their relationship to one another, future research can help bridge the gap between closely related yet fragmented research traditions.

## Supplementary Information

Below is the link to the electronic supplementary material.

Supplementary file1 (DOCX 2784 kb)

## Data Availability

All the data and materials necessary to reproduce the experiment and analyses are available online at: https://doi.org/10.6084/m9.figshare.31026025. None of the study’s analyses were preregistered.

## Code availability

Code is available on request.
