# Supplementary material for: Long-term memory speeds encoding, independently of attention
Source: Psychon Bull Rev. 2026 Sep 8;33(7):229. doi: 10.3758/s13423-026-02998-1 (PMC13553675; doi:10.3758/s13423-026-02998-1)
Supplement: Supplementary file 1 [file 13423_2026_2998_MOESM1_ESM.docx]

**Supplementary Materials**

For this and the following analyses, we coded the targets and distractor categories based on the participants’ language, labelling them as familiar and unfamiliar accordingly.

In RSVP, unencoded targets are at risk of being overridden by temporally adjacent distractors. Thus, focusing on the likelihood of erroneously reporting distractors that follow the target (distractor intrusions) provides another avenue to test the notion that familiarity speeds WM encoding. In Experiments 1-2 (where distractor intrusions could be isolated from other errors), the familiarity effects on accuracy were mirrored by familiarity effects on distractor intrusions, *t*(39) = 3.00, *p* = .005, *d* = 0.48, and *t*(19) = 2.77, *p* = .01, *d* = 0.62, respectively.

To further examine the role of post-target distractors on target reports, we examined whether the familiarity effects are modulated by the category of the post-target distractor, that is, when the distractor was familiar versus unfamiliar. We therefore entered accuracy in Experiments 1-3 as the dependent variable in a repeated-measures ANOVA with target familiarity and post-target distractor familiarity as within-subject factors.

In Experiment 1, the analysis revealed a non-significant interaction between the two factors, *F*(1,19) = 3.17, *p* = .08, $\eta_{p}^{2}$ = .08. For the sake of completeness, we nevertheless conducted an analysis of target familiarity effects when the post-target distractor was familiar and when it was unfamiliar. When the post-target distractor was unfamiliar, target-familiarity had a significant effect on accuracy, *t*(19) = 4.24, *p* < .001, *d* = 0.67. When the post-target distractor was familiar, the effect of target familiarity was not significant, *t*(19) = 1.74, *p* = .09, *d* = 0.28.

In Experiment 2, the analysis revealed a significant interaction between the two factors, *F*(1,19) = 9.31, *p* = .007, $\eta_{p}^{2}$ = .33. When the post-target distractor was unfamiliar, target-familiarity had a significant effect on accuracy, *t*(19) = 4.51, *p* < .001, *d* = 1.01. When the post-target distractor was familiar, the effect of target familiarity was not significant, *t*(19) = -0.24, *p* = .82, *d* = -0.05.

The results from Experiments 1-2 suggest that accuracy is dependent, to some extent, on both the target’s familiarity and the distractor’s familiarity. A familiar target is resilient to being overridden by an unfamiliar distractor, whereas an unfamiliar target is not. Meanwhile, neither a familiar target nor an unfamiliar target is resilient to being overridden by a familiar distractor.

***Supplementary Table 1***. Accuracy In Experiments 1-3.

|  | Unfamiliar Post-target Distractor | | Familiar Post-target Distractor | |
| --- | --- | --- | --- | --- |
|  | Unfamiliar target | Familiar target | Unfamiliar target | Familiar target |
| Experiment 1 | 62.4% | 74.1% | 64.4% | 69.3% |
| Experiment 2 | 62.7% | 79.3% | 62.7% | 61.7% |
|  |  |  |  |  |
|  | Mixed-language Block | | Same-language Block | |
|  | Unfamiliar target | Familiar target | Unfamiliar target | Familiar target |
| Experiment 3 | 71.5% | 64.6% | 57.3% | 67.6% |
